# *Salmonella*-liberated dietary L-arabinose promotes expansion in superspreaders

**DOI:** 10.1016/j.chom.2023.01.017

**Published:** 2023-03-08

**Authors:** Sarah J. Ruddle, Liliana M. Massis, Alyssa C. Cutter, Denise M. Monack

**Affiliations:** 1 Department of Microbiology and Immunology, Stanford University School of Medicine, Stanford, CA 94305, USA

**Keywords:** enteric pathogen, L-arabinose, glycoside hydrolase, alpha-N-arabinofuranosidase, superspreader, Salmonella, fiber

## Abstract

The molecular understanding of host-pathogen interactions in the gastrointestinal (GI) tract of superspreader hosts is incomplete. In a mouse model of chronic, asymptomatic *Salmonella enterica* serovar Typhimurium (*S*. Tm) infection, we performed untargeted metabolomics on the feces of mice and found that superspreader hosts possess distinct metabolic signatures compared with non-superspreaders, including differential levels of L-arabinose. RNA-seq on *S*. Tm from superspreader fecal samples showed increased expression of the L-arabinose catabolism pathway *in vivo*. By combining bacterial genetics and diet manipulation, we demonstrate that diet-derived L-arabinose provides *S*. Tm a competitive advantage in the GI tract, and expansion of *S*. Tm in the GI tract requires an alpha-*N*-arabinofuranosidase that liberates L-arabinose from dietary polysaccharides. Ultimately, our work shows that pathogen-liberated L-arabinose from the diet provides a competitive advantage to *S*. Tm *in vivo*. These findings propose L-arabinose as a critical driver of *S*. Tm expansion in the GI tracts of superspreader hosts.

## Highlights

• *S.* Tm superspreader mice have distinct gut metabolomes compared with non-superspreaders
• *S.* Tm uses L-arabinose to expand in the GI tract
• *S.* Tm acquires dietary L-arabinose through an alpha-*N-*arabinofuranosidase (STM0148)
• Acquisition of L-arabinose is important for the emergence of *S.* Tm superspreader mice

## Graphical abstract

**Figure:**
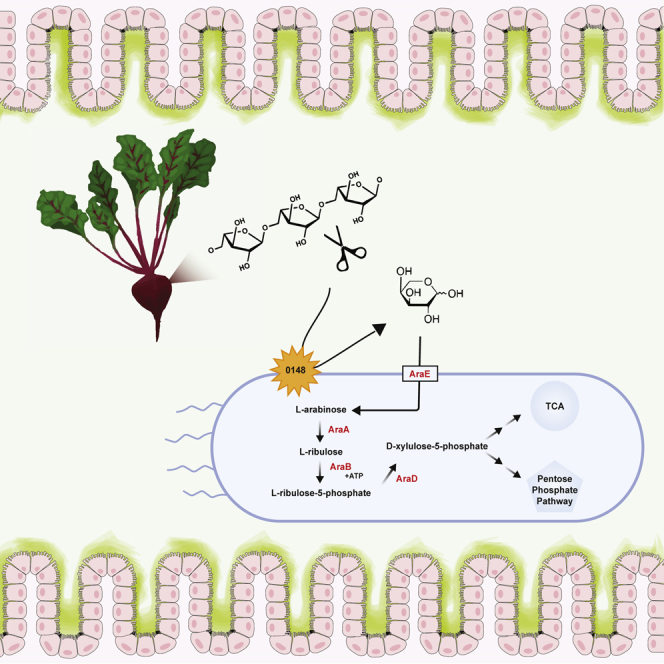

## Teaser

Ruddle et al. combine metabolomics and transcriptomics to show that the pathogen *Salmonella* Typhimurium catabolizes L-arabinose in the GI tracts of superspreader mice. They demonstrate that *S*. *Tm* uses an alpha-*N*-arabinofuranosidase to acquire dietary L-arabinose and find this process critical for the emergence of superspreaders.

## Introduction

*Salmonella enterica* serovars are responsible for millions of infections worldwide. Disease states range from self-limiting gastroenteritis to invasive enteric fever, the latter of which is caused by human-adapted *S. enterica* serovar Typhi (*S.* Typhi).^1^ In mouse oral infection models, *S. enterica* serovar Typhimurium (*S*. Tm) robustly colonizes the cecum and colon.^2^ From these sites *S*. Tm is shed via the feces into the environment, where it can infect naive hosts.^3^ Transmission efficiency has been linked to increased levels of fecal bacteria, with high-shedding hosts (referred to as superspreaders) responsible for most transmission events.^4^ A subset of infected individuals become carriers and persistently shed *Salmonella* in their feces, thereby functioning as a reservoir for the pathogen. The most famous superspreader, Mary Mallon, is remembered by the moniker Typhoid Mary for the dozens whom she infected with *S.* Typhi as a cook at the turn of the century.^5^^,^^6^ For transmission events to occur, *Salmonella* utilizes several key virulence factors such as *Salmonella* pathogenicity islands (SPI-1), SPI-2, and SPI-6, which encode secretion systems that deliver effector proteins into host cells and competing Enterobacteriaceae to establish itself in the gastrointestinal (GI) tract and replicate to high levels while concurrently competing against commensal microbiota for resources.^2^^,^^7^ Although microbiome-*Salmonella* interactions have been shown to influence *Salmonella*-shedding levels,^8^ specific features that distinguish superspreaders from non-superspreaders remain unknown.

The complex microbial community occupying the GI tract plays a crucial role in preventing pathogen invasion through direct and indirect mechanisms, referred to as colonization resistance.^9^ We and others have demonstrated that the commensal microbiota provide colonization resistance against *S*. Tm infection, as oral antibiotic treatment enhances pathogen expansion and fecal shedding.^10^^,^^11^ Mechanisms of colonization resistance against intestinal *S*. Tm infection have mainly been studied in murine colitis models of infection, where mice are pre-treated with antibiotics resulting in high levels of inflammation.^10^ Multiple studies have also used this colitis model to shed light into metabolic pathways that *S*. Tm uses to expand in the GI tracts of antibiotic-treated mice.^8^ For example, *S*. Tm takes advantage of nitrate generated by the host immune response by using it as an electron acceptor to fuel anaerobic respiration,^12^ which allows *S*. Tm to outgrow the resident microbiota.^13^^,^^14^ The inflamed GI tract is a unique niche in which *S*. Tm alters its metabolism and begins to utilize carbon sources that require respiration such as ethanolamine, fructose-asparagine, 1,2-propanediol, and propionate.^15^^,^^16^^,^^17^^,^^18^ Although important discoveries were made, disruption of the resident microbial community in this antibiotic-treated mouse model limits the generalizability of mechanistic findings to clinical infections in humans, in which some individuals become chronic asymptomatic superspreaders. Thus, in this study, we use a mouse model of oral *S*. Tm infection in which mice are not pre-treated with an antibiotic to uncover mechanisms of *Salmonella* colonization that promote the emergence of superspreaders.

In a conventional mouse model, *Salmonella* must directly compete with commensal microbiota for a nutritional niche.^19^ Diets contain complex carbohydrates, the main component of dietary fiber, and are the primary metabolic input for both commensal and pathogenic bacteria.^20^ The ability of commensal microbes to process complex carbohydrates is critical for their colonization of the GI tract. Commensal bacteria process complex carbohydrates through glycoside hydrolases that cleave specific glycosidic linkages to liberate oligosaccharides or monosaccharides for catabolism.^21^ Some bacteria have evolved to sequester free saccharides from competing microbes through mechanisms of rapid import systems,^22^ while others can work in unison to break down complex carbohydrates in so-called cross-feeding events.^23^ *Salmonella* can participate in cross-feeding by catabolizing commensal-liberated fucose and sialic acid from host-derived mucus for its colonization in colitis models.^24^ Although these studies have advanced our understanding of dietary carbohydrate processing by commensals, little is known about how pathogens such as *Salmonella* utilize complex sugars during infection and how these metabolic processes affect superspreaders.

The metabolic networks of host, microbiome, and pathogen are interconnected during infection, and a deeper understanding of the metabolic signatures that underlie superspreaders may provide insights relevant for infection control. In this study, we performed untargeted metabolomic analysis in superspreader and non-superspreader mice to identify a metabolic state that is unique to superspreaders. We narrowed our focus to pathogen-specific factors by analyzing the transcriptional profile of *S*. *Tm* from superspreader feces and identified L-arabinose metabolism as a critical pathway for *Salmonella* expansion in the GI tract. We further found that exogenous supplementation of dietary L-arabinose promotes the rapid emergence of superspreaders. We demonstrate that L-arabinose catabolism confers a strong competitive advantage for *Salmonella* in the GI tract, which was independent of the microbiome. Finally, we identified a previously uncharacterized alpha-*N*-arabinofuranosidase in *Salmonella* that plays a key role during colonization of the GI tract. Collectively, these findings uncover a metabolite-dependent mechanism of pathogen expansion within a mouse model of chronic *Salmonella* infection.

## Results

### *Salmonella* superspreaders have a distinct metabolic signature

**Figure 1:**
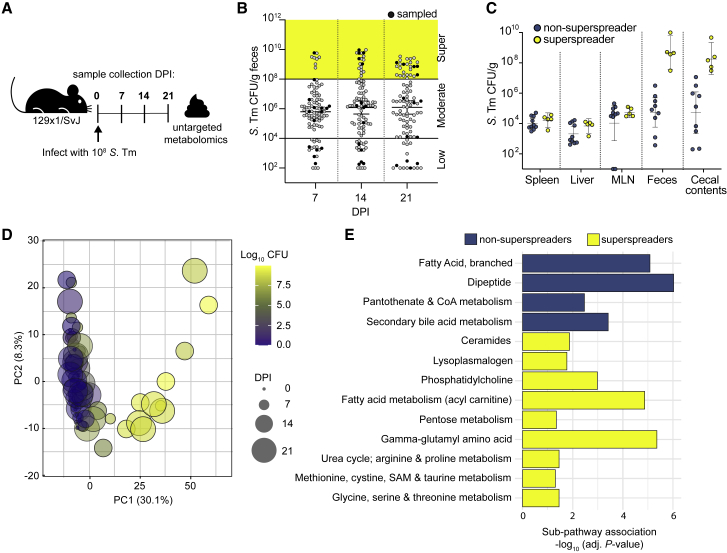
*S.* Tm superspreaders share a distinct metabolic landscape (A) Infection schematic for metabolomic profiling. 129X1/SvJ mice infected with 10^8^ CFU *S*. Tm for untargeted fecal metabolomics. Mice were the sampled at the indicated time points: 0, 7, 14, and 21 days post-infection. (B) *S*. Tm CFU/g feces from 129X1/SvJ 7, 14, and 21 days post-infection (n = 95). Black circles indicate mice selected from each shedding stratum (low, moderate, supershedding; n = 5) for fecal metabolite profiling. (C) S. Tm burden across organs of 15 mice selected for fecal metabolomics day 28 post-infection. n = 5 mice per shedding strata group. (D) Principal component analysis of fecal metabolomes from 20 mice, over 4 time points, colored by CFU, and sized by day post-infection (0–21 DPI). (E) Differential pathway enrichment of non-superspreaders and superspreaders at 14 DPI with multiple hypothesis correction (likelihood ratio test with Benjamini and Hochberg correction). See also Figure S1.

The molecular mechanisms that impact *Salmonella* expansion in the distal GI tracts of chronically infected mice are incompletely understood. To gain insight into mechanisms of pathogen expansion, ninety-five 129X1/SvJ mice were intragastrically inoculated with the wild-type (WT) *S*. Tm strain SL1344, and bacterial loads were quantified in the feces at 7, 14, and 21 days post-infection (DPI) (Figures 1A and S1A). As previously published, mice shed a range of *S*. Tm in the feces over time with approximately 20% of mice shedding >10^8^ colony-forming units (CFUs)/g feces, which we define as superspreaders^4^ (Figure 1B). In addition, spleen, liver, mesenteric lymph nodes (MLNs), cecal contents, and feces were collected 28 DPI to quantify *S*. Tm levels at these different infection sites for comparison to fecal shedding levels. Although the pathogen burden in the feces varied between the mice from the level of detection to 10^9^ CFU/g feces, there were no significant differences in the levels of *S*. Tm in the systemic tissues (Figure 1C). The heterogeneity of *S*. Tm levels was restricted to the distal GI tract,^25^ suggesting that distal GI-tract-specific metabolites may affect pathogen expansion.

To better understand how the metabolic landscape of the intestine changes during *S*. Tm infection, we performed unbiased, semi-quantitative profiling on extracellular metabolites in the GI tract over time. Specifically, fecal samples from mice shedding low (10^2^–10^4^ CFU/g), moderate (10^4^–10^8^ CFU/g), or superspreader (>10^8^ CFU/g) levels of pathogen (n = 5 per shedding group; Figure 1A) were collected and subjected to ultra-high-performance liquid chromatography-tandem mass spectrometry (UHPLC/MS). The relative abundances of 797 metabolites in the feces were measured in infected mice over time and compared with mock-treated mice (Metabolon, USA). Analysis of the longitudinal samples identified a distinct metabolic signature that developed in superspreaders over time (Data S1). Principal component analysis (PCA) of the full dataset showed the effect of *S*. Tm burden (log CFU) on the metabolome was largely captured by PC1 (Figure 1D), with the first two PCs accounting for 38.4% of the variation in the dataset. PCA showed partitioning by shedding stratum (Figure 1D), where superspreaders clustered together and away from low and moderate shedders, indicating superspreaders share a distinct metabolome compared with the low and moderate shedders.

Pathway enrichment analysis identified metabolic pathways with significant differential enrichment in superspreader and non-superspreader mice throughout the infection (Figures 1E, S1B, and S1C, likelihood ratio test with Benjamini & Hochberg correction). Acyl-carnitine, pentose, gamma-glutamyl amino acid, and phosphatidyl choline metabolism are among the pathways enriched in superspreader versus non-superspreader feces on 14 DPI (Figure 1E). Enriched pathways of non-superspreader feces collected on 14 DPI include secondary bile acid, dipeptide, and branched fatty acid metabolism pathways (Figure 1E). Comparing pathway enrichment across time points highlights the metabolic flux occurring over the time course of infection. For example, acyl-choline and tocopheral metabolism are enriched in superspreader mice versus non-superspreader mice only at 7 DPI (Figure S1B). In contrast, metabolic pathways like ceramides associate with superspreaders at 14 and 21 DPI (Figures 1E, S1B, and S1C). Together, these findings suggest that the metabolic landscape of superspreader mice is distinct and may reflect alterations in luminal nutrient availability during *S*. Tm infection.

### *S*. *Tm* gene expression in GI tracts of superspreader mice

**Figure 2:**
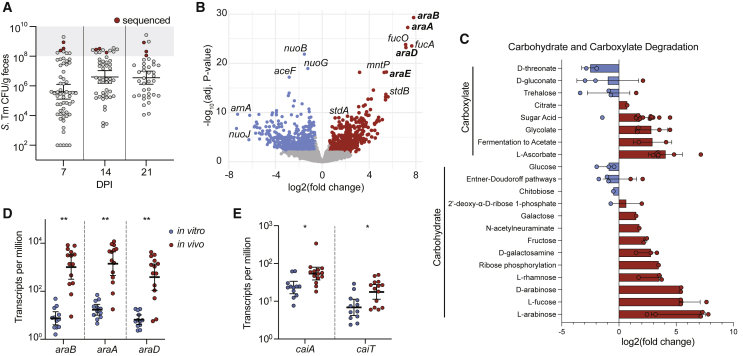
*In vivo* fecal transcriptomics of *S*. Tm superspreaders reveal changes in carbohydrate utilization (A) 129X1/SvJ mice infected with 10^8^ CFU WT *S*. Tm for *in vivo* RNA-seq. *S*. Tm shedding levels over time, red data points indicate superspreader mice selected for metatranscriptomic analysis of feces on 7, 14, and 21 DPI. n = 3 at each time point. (B) Significant differentially expressed genes *in vitro* (blue) and *in vivo* (red). Non-significantly enriched genes in gray, −log_10_ adjusted p value and log_2_ fold change. (C) Log_2_ fold change of differentially enriched metabolites (adjusted p value < 0.05; Benjamini and Hochberg correction) *in vitro* (blue) and *in vivo* (red). Each data point represents single metabolites within each sub-pathway (y axis). Sub-pathways are annotated with corresponding major pathways (brackets along y axis). (D) Transcripts per million of *araB*, *araA*, and *araD in vitro* (blue) and *in vivo* (red). ^∗∗^p < 0.01 t test with Welch’s correction. (E) Transcripts per million of *caiA* and *caiT in vitro* (blue) and *in vivo* (red). ^∗^p < 0.05 t test with Welch’s correction. See also Figure S2.

Next, we performed metatranscriptomic analysis to prioritize *S*. Tm phenotypes that support expansion in the distinct metabolic environment of superspreaders. Metatranscriptomics quantifies mRNA across the GI microbial community and can demonstrate which genes are differentially expressed between contexts. Mice were infected with WT *S*. Tm strain SL1344 (*S*. Tm^WT^), and fecal shedding levels were monitored over time (Figure 2A). After 7, 14, and 21 DPI, mice were euthanized, and cecal contents and feces were collected for total RNA extraction (including host, commensal microbes, and *S*. Tm RNA). Three superspreader mice at each time point were used for bulk *in vivo* RNA-seq (Figure 2A). A technical limitation of *in vivo* RNA-seq of a mixed microbial community prevents robust detection of bacterial species present at low abundances. Previous work shows that the microbe of interest should be present at approximately 1% of the total community to recover an appropriate number of reads for downstream analysis. Due to this limitation, RNA-seq was only done in mice harboring over 10^8^ CFU/g *S*. Tm (superspreaders). A range of 879,396–2,209,413 reads per *in vivo* sample aligned to the reference *S*. Tm genome (0.94%–3.05% total reads). In addition to the *in vivo* samples, RNA was extracted from *S*. Tm grown in LB cultures, grown both aerobically and anaerobically to mid-log, log, and stationary stage. PCA analysis comparing *S*. Tm transcriptomes of all *in vitro* versus all *in vivo* conditions showed that the *in vivo* data separated from *in vitro* samples along PC1 (Figure S2A). We found 1,755 differentially expressed genes between *in vivo* (cecum and feces) and *S*. Tm grown *in vitro* (adjusted p < 0.05, likelihood ratio test with Benjamini & Hochberg correction).

Genes involved in several metabolic pathways were enriched in *S*. Tm from superspreader mice compared with *in vitro* samples (Figure 2B). Genes that encode known virulence factors, such as *ssaV*, *sifA*, *pipB2*, and *sseI*, were expressed at lower levels in the GI tract compared with *in vitro* growth conditions, which is consistent with the expression of these virulence factors being important for *S*. Tm growth and survival in tissues rather than growth in the gut lumen.^26^^,^^27^ In contrast, genes previously demonstrated to impact *Salmonella* colonization of the GI tract, such as fucose metabolism (*fucA*, *fucO*, *fucK*)^24^^,^^28^ and fimbriae genes (*stdA, stdB*)^29^^,^^30^ were expressed at higher levels in the GI tracts compared with *in vitro* growth conditions (Figure 2B). We also plotted the log_2_ fold change of all significantly enriched genes by their functional pathway to visualize an overview of transcriptional changes *S*. Tm undergoes in superspreader hosts versus *in vitro* growth conditions (Figure S2B). We observed overlapping enriched pathways from the transcriptomics and the metabolomics. For example, gene transcripts from the carbohydrate and carboxylate degradation pathways were enriched *in vivo* (Figure S2B), and changes in pentose metabolites were enriched in superspreaders (Figure 1E). To look closer at carbohydrate catabolism dynamics in *S*. Tm, we plotted the log_2_ fold change of genes involved in the carbohydrate and carboxylate degradation pathways and found L-arabinose degradation to be the most enriched pathway *in vivo* (Figure 2C). Interestingly, glucose degradation gene transcripts were lower *in vivo* than those *in vitro*, which agree with the cross regulation of L-arabinose and glucose utilization in *S*. Tm.^31^ Transcripts from the L-arabinose utilization operon (*araB*, *araA*, and *araC*) isolated from *S*. Tm *in vivo* were ∼100-fold higher compared with *in vitro* conditions (Figure 2D).

In addition to L-arabinose catabolism, levels of the carnitine catabolism gene transcripts (*caiA*, *caiT*) were enriched in *S*. Tm isolated from superspreader cecum and feces compared with *S*. Tm grown *in vitro* (Figure 2E). The metabolomics data also indicated that superspreaders have a unique carnitine metabolic profile, as the acyl-carnitine pathway was enriched in superspreaders (Figure 1E). Together, these data suggest that *S*. Tm catabolism of L-arabinose and carnitine may play roles in colonization of superspreader GI tracts.

### L-arabinose and carnitine metabolism changes in superspreader mice

**Figure 3:**
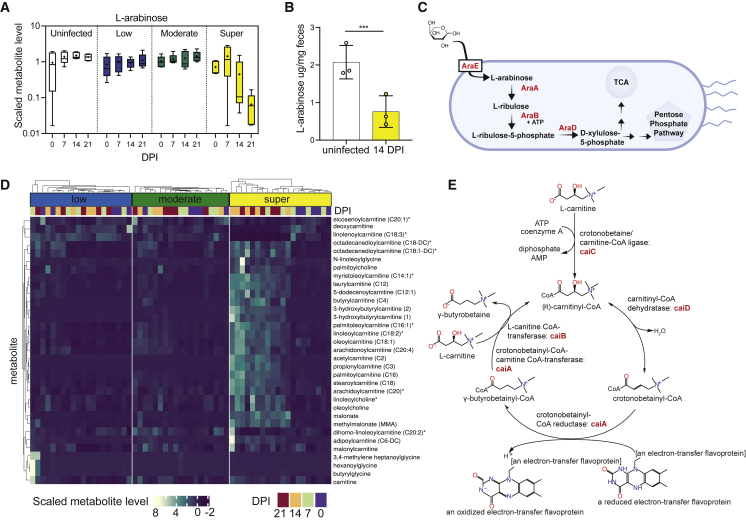
L-arabinose metabolism is important for *S*. Tm expansion in superspreader mice (A) Scaled-metabolite level of L-arabinose detected in stool of uninfected and infected mice colonized across shedding strata. (B) L-arabinose levels detected in uninfected mice and superspreader mice feces (collected 14 DPI) by targeted LC/MS. t test ^∗∗∗^p < 0.001. (C) Simplified schematic of L-arabinose catabolism in *Salmonella enterica*. (D) Heatmap showing scaled-metabolite levels of metabolites from carnitine metabolism pathway for all shedding strata and four time points (0–21 DPI). (E) Schematic of L-carnitine catabolism in *S*. Tm.

The increased expression L-arabinose and carnitine metabolism genes from *S*. Tm isolated from superspreader mice led us to focus on the roles of these pathways *in vivo*. We observed significant differences in the pentose metabolism and acyl-carnitine sub-pathways in superspreaders compared with non-superspreaders (Figure 1E). The scaled level of L-arabinose decreased in superspreader mice over time, yet it remained constant across uninfected, low, and moderate shedders (Figure 3A). We confirmed the levels of L-arabinose by quantifying the L-arabinose/g feces of uninfected mice and superspreader mice at 14 DPI through targeted LC/MS. Similar to the scaled-metabolite levels, there were significantly lower levels of L-arabinose in the feces of superspreader mice compared with uninfected mice (Figure 3B). These data led us to hypothesize that *S*. Tm consumes L-arabinose in the gut lumen of superspreader mice. Although L-arabinose metabolism in *Enterobacteriaceae* has been relatively well characterized,^32^^,^^33^ the role of L-arabinose metabolism during infection has not been studied in detail. In *Salmonella enterica* serovars, the primary L-arabinose permease is AraE.^34^ Intracellularly, the ribulokinase, AraB, and the L-arabinose isomerase, AraA, produce L-ribulose-5-phosphate from L-arabinose. AraD, an L-ribulose-5-phosphate 4-epimerase, then forms L-xylulose-5-phosphate. L-xylulose-5-phosphate is further processed in established downstream pathways ultimately feeding into the pentose phosphate pathway or the TCA cycle^35^ (Figure 3C). Most metabolites within the acyl-carnitine pathway increased in superspreader mice over time, yet they remained constant across low and moderate shedders (Figure 3D). Upon import into the cell through CaiT, the L-carnitine:gamma-butyrobetaine antiporter, *S*. Tm can degrade L-carnitine as outlined in Figure 3E.^36^ The metabolite level and gene expression changes of carnitine derivatives led us to question whether *S*. Tm could benefit from their surplus in the GI environment.

### L-arabinose catabolism confers competitive advantage to *S*. Tm in the GI tract

**Figure 4:**
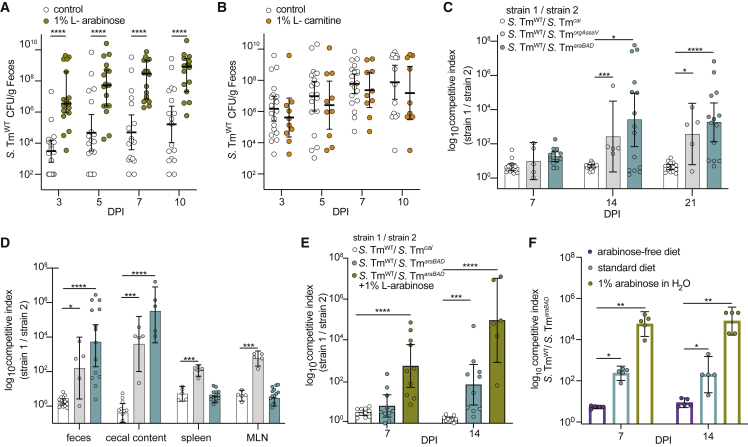
L-arabinose utilization confers a competitive advantage to *S*. Tm *in vivo* (A) 129X1/SvJ mice infected with *S*. Tm^WT^ (SL1344) and CFU/g feces quantified over 10 days. White dots indicate control mice (n = 18) and green dots represent mice provided 1% exogenous L-arabinose through drinking water (n = 18). ^∗∗∗^p < 0.001, ^∗∗∗∗^p < 0.0001 Multiple Mann-Whitney tests. (B) 129X1/SvJ mice infected with WT *S*. Tm^WT^ (SL1344) and CFU/g feces quantified over 10 days. White dots indicate control mice (n = 20) and orange dots represent mice provided 1% exogenous L-carnitine through drinking water (n = 10). No significant difference between groups by multiple Mann-Whitney tests. (C) 129X1/SvJ mice were inoculated with an equal mixture of the indicated strains. The competitive index of the two strains in the feces was determined at 3, 7, 14, and 21 days post-infection. (D) 129X1/SvJ mice (from Figure 4A) were euthanized 21 DPI for organ collection. Spleen, mesenteric lymph nodes, cecal content, and feces were homogenized and plated for CFU and competitive index. (E) 129X1/SvJ mice were inoculated with an equal mixture of the indicated strains. One group of mice were provided 1% L-arabinose drinking water (green). The competitive index in the stool was determined on 7 and 14 days post-infection. n = 10 mice per group. (F) Germ-free mice were given the indicated dietary conditions one day prior to infection. Mice were infected with an equal mixture of *S*. Tm^*orgAssaV*^ and *S*. Tm^*orgAssaVaraBAD*^. Competitive index was determined at 7 and 14 days post-infection. n = 5 per group. Each dot represents data from one animal (biological replicate). Scale bars represent ± SEM. ^∗^p < 0.05, ^∗∗^p < 0.01, ^∗∗∗^p < 0.001, ^∗∗∗∗^p < 0.0001. Mann-Whitney U test unless otherwise stated. See also Figures S3 and S4.

The overlap of our metabolomic and transcriptomic data in superspreaders led us to hypothesize that *S*. Tm exploits L-arabinose and/or L-carnitine to expand in the distal GI tract. To interrogate the importance of these pathways, we provided 1% exogenous L-arabinose or 1% L-carnitine in the drinking water of mice, concentrations sufficient to impart phenotypes in previous rodent studies,^37^^,^^38^^,^^39^ at the start of infection and maintained mice on the treated water throughout the course of infection. Strikingly, *S*. Tm expanded rapidly in the GI tracts of mice provided exogenous L-arabinose, where the average shedding levels in mice provided exogenous L-arabinose were >100-fold higher than control mice by 3 DPI (Figure 4A). In contrast, mice given exogenous L-carnitine shed similar levels of *S*. Tm compared with mice given control (standard) water throughout the course of infection (Figure 4B). The dramatic expansion of *S*. Tm in mice supplemented with L-arabinose led us to question the indirect effect exogenous L-arabinose could be having in the mice. Inflammation has been well established to facilitate *S*. Tm expansion in the GI tract by various mechanisms that include use of an array of exogenous respiratory electron acceptors, consumption of fermentation products, or oxidized sugars.^40^ To determine whether exogenous L-arabinose was creating an inflammatory environment for *S*. Tm to exploit, we profiled fecal levels of inflammatory marker lipocalin-2 and proinflammatory cytokine mRNA levels in the presence or absence of exogenous L-arabinose from uninfected and *S*. Tm^WT^ infected mice. There was no significant difference in lipocalin-2 levels (Figure S3A) in the feces or mRNA transcripts of inflammatory cytokines interferon gamma, tumor necrosis factor-alpha, interleukin-1 beta, or interleukin-6 in the colon or spleen (Figures S3B and S3C). Together these data indicate that *S*. Tm uses L-arabinose to expand in the GI tract, independent of inflammation. Ruling out indirect inflammation brought on by increased exogenous L-arabinose led us to hypothesize that arabinose is an important carbon source for *S*. Tm in the GI tract.

To test whether L-arabinose utilization or carnitine degradation pathways confer a fitness advantage for *S*. Tm in the GI tract, we infected mice with an equal mixture of WT *S*. Tm and mutants and calculated a competitive index (CI) at multiple time points throughout the course of infection. Groups of 129X1/SvJ mice were orally inoculated with an equal mixture of the *S*. Tm^WT^ strain and either an L-arabinose catabolism mutant strain, Δ*araBAD::Kan*^*R*^ (*S*. Tm^*araBAD*^), or a carnitine catabolism mutant strain, Δ*caiTABCD::Kan*^*R*^ (*S*. Tm^*cai*^). As a positive control, mice were orally infected with an equal mixture of WT *S*. Tm and a strain that is deficient for critical components of the type-three secretion systems (T3SS) in *S*. Tm, Δ*orgA*Δ*ssaV::Kan*^*R*^ *S*. Tm (*S*. Tm^*orgAssaV*^), and is attenuated for colonization of conventional mice.^11^ As expected, *S*. Tm^WT^ outcompeted *S*. Tm^*orgAssaV*^, with CI of ∼100 at 14 DPI (Figure 4C). In contrast, the *S*. Tm^WT^ and *S*. Tm^*cai*^ strains were equally fit during the 21-day infection (Figure 4C). Since the L-carnitine-deficient mutant did not have a defect in the GI tracts of mice, we utilized this mutant as a control for the chromosomal insertion of the kanamycin resistance gene in subsequent competition experiments. We found that the *S*. Tm^*araBAD*^ strain suffered a competitive disadvantage after 14 DPI compared with *S*. Tm^WT^, with a CI of >1,000 in the GI tracts of mice (Figure 4C), which was even higher than the fitness defect observed with the control T3SS-deficient strain. Collectively, our data indicate that L-arabinose metabolism provides a competitive advantage to *S*. Tm in the GI tract.

To determine whether *S*. Tm utilization of L-arabinose confers a competitive advantage in other tissues, we measured *S*. Tm levels in cecal content, MLNs, and spleen at 21 DPI. The *S*. Tm^WT^ strain outcompeted the *S*. Tm^*araBAD*^ strain in the cecal contents (Figure 4D). In contrast, the *S*. Tm^WT^ and *S*. Tm^*araBAD*^ strains were equally fit in the MLN and spleen (Figure 4D), indicating that L-arabinose metabolism confers a competitive advantage to *S*. Tm specifically in the GI tract.

These results led us to hypothesize that dietary L-arabinose supplementation would lead to greater competition between the WT and the L-arabinose catabolism-deficient *S*. Tm mutant strains. To evaluate this hypothesis, two groups of 129x1/SvJ mice were infected with a 1:1 mixture of *S*. Tm^WT^ and *S*. Tm^*araBAD*^ strains. One of these two groups received 1% exogenous L-arabinose in the drinking water at the time of infection and throughout the infection. We found that the kinetics by which the WT *S*. Tm strain outcompeted the *S*. Tm^*araBAD*^ strain were significantly faster in mice given L-arabinose in their water (Figure 4E). Specifically, *S*. Tm^WT^ outcompeted the *S*. Tm^*araBAD*^ strain as early as 7 DPI with a CI∼1,000, and by 2 weeks the CI was ∼10,000 (Figure 4E). Together, these results are consistent with a model in which L-arabinose is a carbon source available to *S*. *Tm* and its catabolism through AraBAD contributes to GI tract colonization and expansion.

In parallel, we assessed whether exogenous L-carnitine would lead to a competitive advantage for WT *S*. Tm compared with *S*. Tm^*cai*^. Mice were given standard water or 1% L-carnitine water throughout the course of infection. To ensure mice were ingesting the 1% L-carnitine water, we gavaged additional groups of mice daily with 200 μL of standard water or 1% L-carnitine water. There was no competitive advantage to WT *S*. Tm in any of the tested conditions (Figure S4A). Together these data suggest that *S*. Tm does not directly benefit from L-carnitine degradation during chronic mouse infection. With a CI of ∼1, *S*. Tm^*cai*^ continues to serve as a control for the chromosomal insertion of the kanamycin resistance gene. Moving forward, we chose to continue probing mechanisms of L-arabinose metabolism during *S*. Tm infection.

### Competitive advantage of *S*. Tm L-arabinose catabolism in the GI tract is not dependent on commensal bacteria

Given the critical role that L-arabinose utilization plays during *S*. Tm colonization of the GI tract, we next sought to understand how *S*. Tm acquires L-arabinose in the gut lumen of superspreader mice. In mice, L-arabinose is found within plant polysaccharides of fibrous dietary components and requires microbe-specific enzymes, glycoside hydrolases, to liberate free L-arabinose^41^. To determine if the microbiota was responsible for liberating free L-arabinose in the GI lumen for subsequent use by *S*. Tm during infection, we performed a series of CI experiments in germ-free mice. 1 day prior to infection, germ-free mice were given either a standard diet and standard water, an arabinose-free diet and standard water, or standard diet and water containing 1% L-arabinose. WT *S*. Tm is lethal to germ-free mice,^42^ and both T3SSs must be functionally inactive for *S*. Tm to colonize the mouse GI tract while maintaining host viability.^43^ Thus, to assess *S*. Tm fitness and metabolism in the absence of other commensal microbes, germ-free mice were infected orally with a 1:1 mixture of *S*. Tm^*orgAssaV*^ and *S*. Tm^*orgAssaVaraBAD*^ strains. In mice fed an arabinose-free diet, the *S*. Tm^*orgAssaV*^ strain had no competitive advantage compared to the L-arabinose-utilization mutant strain, *S*. Tm^*orgAssaVaraBAD*^ (Figure 4F). In contrast, the *S*. Tm^*orgAssaV*^ strain was significantly more fit than the *S*. Tm^*orgAssaVaraBAD*^ strain in mice given standard chow and standard water, which contains L-arabinose in the form of crude plant material (dietary fiber) (Figure 4F). Furthermore, we found that the *S*. Tm^*orgAssaV*^ strain outcompeted the *S*. Tm^*orgAssaVaraBAD*^ strain more rapidly and significantly in mice fed a standard diet and 1% L-arabinose water (Figure 4F). We also confirmed through LC/MS, that L-arabinose levels decrease during *S*. Tm colonization in the gnotobiotic (ex-germ-free), similar to our findings in the conventional 129X1/SvJ mouse model (Figure S4B). Together these data reveal that *S*. Tm has a competitive advantage when able to catabolize L-arabinose, even in the absence of a commensal microbiota.

### *S*. Tm arabinofuranosidase confers competitive advantage to *S*. Tm in the GI tract

**Figure 5:**
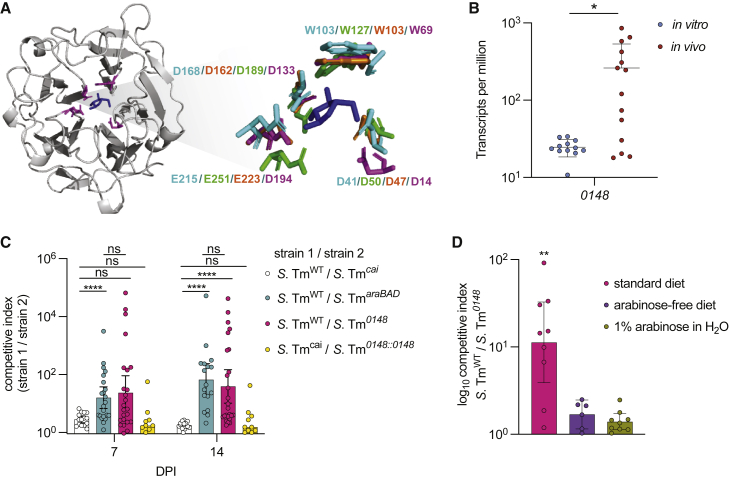
*S.* Tm uses a functionally conserved arabinofuranosidase to colonize the GI tract (A) Predicted structure of STM0148 generated with Phyre. Critical residues highlighted in purple surround L-arabinose. Call-out shows structural alignment of critical residues from *S*. Tm (in purple) with characterized arabinofuranosidase enzymes from *Cellvibrio japonicus* (PDB accession 3QEE) in teal, *Bacillus subtilis* in green (PDB accession 3C7G), and *Streptomyces avermitilis* (PDB accession 3AKH) in orange. L-arabinose molecule is derived from the *S. avermitilis* structure (3AKH). (B) *Salmonella* Typhimurium putative arabinofuranosidase (STM0148) is differentially expressed *in vivo* compared with *in vitro*. t test ^∗^p < 0.05. (C) 129X1/SvJ mice were inoculated with an equal mixture of the indicated strains. The competitive index from the feces was determined at 7 and 14 days post-infection. Each dot represents data from one animal (biological replicate). Scale bars represent ± SEM. ^∗^p < 0.05, ^∗∗^p < 0.01, ^∗∗∗^p < 0.001, ^∗∗∗∗^p < 0.0001. Mann-Whitney U. (D) 129X1/SvJ mice were inoculated with an equal mixture of *S*. Tm^WT^ and *S*. Tm^*0148*^. Exogenous L-arabinose was provided in the drinking water of the indicated group shown in green, and an arabinose-free diet was given to the indicated group shown in purple. The competitive index was determined at 7 DPI. Each dot represents data from one animal (biological replicate). Scale bars represent ± SEM. ^∗^p < 0.05, ^∗∗^p < 0.01. Kruskal-Wallis.

Since the increased fitness of *S*. Tm conferred by L-arabinose catabolism is not dependent on the microbiome, we hypothesized that *S*. Tm has the functional capacity to liberate free L-arabinose from plant polysaccharides for its own use *in vivo*. To test this hypothesis, we first performed a nucleotide sequence BLAST search using the NCBI database to identify potential arabinose-liberating enzymes in *S*. Tm and found that many *Salmonella enterica* genomes encode an uncharacterized gene with high sequence homology to characterized alpha –*N-* arabinofuranosidases. The carbohydrate-active enzymes database (CAZy; http://www.cazy.org), predicts *S*. Tm to encode a single arabinofuranosidase of the 48 total predicted glycoside hydrolases in *S*. Tm SL1344 (Table S1). A multiple sequence alignment program (MAFT) was used to align the *S*. Tm arabinofuranosidase sequence to functionally characterized bacterial arabinofuranosidases (Figure S5A).^44^ The predicted structure of this uncharacterized hydrolase using Phyre revealed that the critical residues meditating substrate binding and enzymatic activity are conserved between this protein and previously characterized alpha –*N-* arabinofuranosidases (Figure 5A). Importantly, our transcriptome data revealed that this putative hydrolase (STM0148) was differentially expressed *in vivo* (Figure 5B), strongly suggesting that *S*. Tm expresses this enzyme during GI tract colonization. We went on to align *S*. Tm SL1344’s alpha –*N-* arabinofuranosidase nucleotide sequence to those of other *Salmonella* serovars. We found that SL1344 shared 100% sequence homology with ST313 isolate *S*. Tm D23580 and *S*. Tm (4, [5],12:i:-) when aligned using MAFT. We identified single-nucleotide polymorphisms (SNPs) when comparing nucleotide sequences of SL1344 with invasive serovars *S.* Dublin, *S.* Paratyphi A, and *S.* Typhi (Ty2). We also aligned the amino acid sequences of all mentioned serovars to the amino acid sequence of SL1344 and found differences in the amino acid sequences of the invasive serovars; however, no residues critical to the function of the alpha-*N-*arabinofuranosidase were altered (Figure S5B).

To test whether the function of this putative arabinofuranosidase contributes to colonization of the GI tract, a strain lacking this gene (*STM0148*) was engineered, Δ*0148::Kan*^*R*^ (*S*. Tm^*0148*^), and used in competition experiments in mice. Mice were maintained on a standard chow that contains L-arabinose-containing plant polysaccharides in the form of crude plant material. *S*. Tm^WT^ was recovered from the GI tracts at significantly higher levels than the *S*. Tm^*0148*^ strain over a time course of infection (Figure 5C), revealing this putative arabinofuranosidase confers a competitive advantage for *S.* Tm *in vivo*. The *S*. Tm^*0148*^ strain was complemented by inserting full length *0148* gene back into the native locus, replacing the kanamycin resistance cassette to generate the *S*. Tm strain Δ*0148::0148* (*S*. Tm^*0148::0148*^). We infected mice with a 1:1 mixture of *S*. Tm^*cai*^ and *S*. Tm^*0148::0148*^ and found the complement strain successfully restored the WT phenotype of the *S*. Tm^*0148*^ *Kan*^*R*^ strain, indicating that the phenotype of the *S*. Tm^*0148*^ strain is due to loss of intact *0148* and not due to potential off-target mutations outside of this locus (Figure 5C).

To further interrogate the role of *S*. Tm’s putative arabinofuranosidase in the acquisition and utilization of L-arabinose *in vivo*, 129X1/SvJ mice fed a polysaccharide-defined diet free of L-arabinose-containing sugars were infected with a 1:1 ratio of *S*. Tm^WT^ and *S*. Tm^*0148*^ strains. *S*. Tm^WT^, and *S*. Tm^*0148*^ strains were recovered at similar levels in the L-arabinose-deficient GI tracts at 7 DPI, revealing that the arabinofuranosidase is not advantageous to *S.* Tm when mice are fed a diet lacking L-arabinose-containing polysaccharides (Figure 5D). The experiment was terminated at 7 DPI due to significant weight loss of mice fed an arabinose-free diet. This finding was similar to a previous study demonstrating deleterious effects of polysaccharide-defined diets lacking fiber, which are exasperated during pathogen infection.^45^ To further probe the function of the arabinofuranosidase *in vivo*, we infected a group of 129X1/SvJ mice fed a standard diet and given water supplemented with 1% L-arabinose with equal concentrations of the *S*. Tm^WT^ and *S*. Tm^*0148*^ strains. In these mice, *S*. Tm^WT^ did not have a competitive advantage against *S*. Tm^*0148*^*,* demonstrating that both the removal of L-arabinose-containing polysaccharides from the diet and the supplementation of free L-arabinose were sufficient to ablate the competitive advantage conferred by the arabinofuranosidase (Figure 5D).

### The emergence of superspreaders depends on the acquisition and utilization of L-arabinose by *S*. Tm

**Figure 6:**
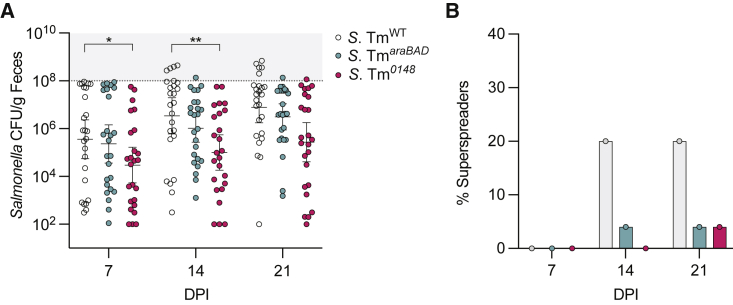
The emergence of superspreaders depends on the acquisition and utilization of L-arabinose by *S*. Tm (A) 129X1/SvJ mice infected with WT *S*. Tm, *S*. Tm^*araBAD*^, or *S*. Tm^*0148*^. S. Tm CFU/g feces quantified over 21 days. WT SL1344 infected mice (n = 25) represented in white, *S*. Tm^*araBAD*^ (n = 25) in blue, and *S*. Tm^*0148*^ (n = 25) in pink. ^∗^p < 0.05, ^∗∗^p < 0.01 Multiple Mann-Whitney tests. (B) Bar graph quantification of percent superspreaders at 7, 14, and 21 DPI. WT infected mice represented in gray bars, *S*. Tm^*araBAD*^ in blue, and *S*. Tm^*0148*^ infected mice in pink.

To determine whether *S*. Tm needs L-arabinose to expand in the GI tract to superspreader levels (>10^8^ CFU/g feces) we infected 129X1/SvJ mice with either *S*. Tm^WT^ or mutants, *S*. Tm^*araBAD*^ or *S*. Tm^*0148*^. Twenty percent of mice infected with *S*. Tm^WT^ became superspreaders by 14 DPI and remained superspreaders on 21 DPI (Figures 6A and 6B). Stinkingly, only 4% of mice infected with *S*. Tm^*araBAD*^ were superspreaders by 14 DPI. The levels of *S*. Tm^*0148*^ shed by infected mice were significantly lower than the *S*. Tm^WT^ strain on 7 and 14 DPI (Figure 6A). None of the 25 mice infected with *S*. Tm^*0148*^ had emerged as superspreaders by 14 DPI and only 4% of mice became superspreaders by 21 DPI (Figure 6B). Collectively, these data demonstrate that *S*. Tm uses a functional alpha-*N*-arabinofuranosidase *in vivo*, where the enzyme liberates free L-arabinose from complex plant polysaccharides, allowing *S*. Tm to expand in the GI tract to superspreader levels (Figure 6C).

## Discussion

Superspreaders are responsible for *Salmonella* transmission events, yet the molecular mechanisms that underlie superspreader development are understudied. The emergence of superspreaders likely depends on a number of complex interactions between host, microbiome, and pathogen that appear to be driven by the metabolic microenvironment of the intestine.^4^^,^^11^^,^^46^ Here, we leveraged a mouse model of chronic *Salmonella* infection in which the gut ecosystem is not perturbed by antibiotics. Using this model, we identified mechanisms by which superspreaders arise in mice infected with *S*. Tm.^4^^,^^47^^,^^48^ The use of unbiased fecal-metabolome mapping of mice harboring heterogeneous levels of *S*. Tm over time allowed us to resolve a metabolic phenotype of *Salmonella* superspreader mice. In parallel, we performed transcriptomics on *S*. Tm recovered from the feces of superspreader mice. By analyzing these two datasets in tandem, we identified L-arabinose metabolism as a critical feature of superspreading mice, revealing that the consumption of this pentose sugar was advantageous for the expansion of *S*. Tm in the GI tract. Collectively, our data shed light on a nutrient-driven mechanism by which *Salmonella* expands within the GI tracts of superspreading mice and highlights the role that diet-derived factors, such as L-arabinose, may play in the emergence of superspreader hosts.

To colonize the GI tract, *Salmonella* must overcome colonization resistance while employing mechanisms of expansion in parallel. In addition to competing with *Salmonella* for carbohydrate sources, commensals can interfere with *Salmonella* expansion through production of short chain fatty acids or antimicrobial peptides.^9^^,^^46^ The microbiota can also indirectly inhibit *Salmonella* through induction of innate and adaptive immune responses.^49^ Concurrently, *Salmonella* has a type VI secretion system it employs for contact-dependent killing of commensals.^7^ *Salmonella* also can exploit commensals by using their fermentation end-product, hydrogen, as an electron donor.^50^ Here, we propose an additional mechanism of pathogen expansion that relies on the selective consumption of a dietary sugar, L-arabinose.

Rolf Freter’s nutrient niche theory claims that a pathogen can only colonize an ecological niche if it is more efficient at using a particular limiting nutrient than its competitors.^51^ It is well established that the ability to degrade carbohydrates, diet or host-mucus derived, provides a competitive advantage to colonizing microbes of the GI tract.^21^^,^^23^ The carbohydrate-active enzymes (CAZymes) involved in the degradation of complex carbohydrates include glycoside hydrolases, polysaccharide lyases, and carbohydrate esterases. CAZymes tend to make up large proportions of GI tract commensal genomes. For example, the common GI tract resident Bacteroides thetaiotaomicron dedicates ∼7% of its genome to glycoside hydrolases.^52^ While CAZymes have been studied in detail within various commensal microbes and marine ecosystems,^53^^,^^54^ the role these enzymes play in pathogenic bacteria are still unclear. Interestingly, chitinases, which hydrolyze chitin polymers into N-acetyl glucosamine oligomers, are a well-studied family of glycoside hydrolases in pathogens. Recent work has characterized the *Salmonella* GH18 family chitinases and have shown them to be important for epithelial adhesion and invasion.^55^^,^^56^ Chitinases have been identified as important virulence factors for several other pathogenic bacterial species including *Legionella pneumophila*,^57^ *Listeria monocytogenes*,^58^ *Vibrio cholerae*,^59^ and Adherent-invasive *Escherichia coli*.^60^ In contrast to chitinases mediating infection as a virulence factor, we propose a mechanism in which *Salmonella* gains a selective advantage through the acquisition of dietary L-arabinose using a previously uncharacterized alpha-*N*-arabinofuranosidase. We show the *Salmonella* alpha-*N*-arabinofuranosidase shares critical residues with functionally characterized arabinofuranosidase enzymes in the glycoside hydrolase family 43 (Figures 5A and 5B).^44^ We also provide evidence that the *Salmonella* alpha-*N*-arabinofuranosidase is functionally active *in vivo* and confers a competitive advantage for *Salmonella* in the GI tract (Figure 5).

Metabolic cross-feeding among members of the microbiota can result in complex food chains, where species may depend on others for partial glycan degradation.^61^ For example, *Bacteroides* species can act as primary degraders of complex carbohydrates, releasing oligosaccharides for secondary degraders to process and consume.^62^ Cross-feeding examples have been described for various dietary glycans including arabinoxylan^63^ and inulin.^64^ *S.* Tm is known to depend on commensal bacteria for liberation of readily consumable monosaccharides like fucose.^24^ We speculate that as *S.* Tm becomes a more dominant member of the community, self-reliance is a failsafe strategy. *S.* Tm using its arabinofuranosidase to acquire L-arabinose may be an example of such an autonomous pathway. This study provides the groundwork for future research to address the role of *Salmonella’s* arabinofuranosidase in the metabolic cross-feeding webs occurring in the GI tract.

An interesting area for future studies will be to understand the regulation of *Salmonella* acquisition of L-arabinose in the GI tract, and what may prevent *Salmonella* from exploiting this nutritional niche in low and moderate shedding mice. Previous work investigating *Salmonella* transcriptional changes during exposure to bile *in vitro* have observed changes in regulation of the arabinose utilization operon.^65^^,^^66^ Interestingly, data from our metabolomics study indicate changes in bile metabolism across shedding groups (Figure 1E). Ongoing experimentation is exploring how alterations in the composition of bile across shedding groups may impact the expression of *Salmonella* arabinose acquisition and utilization genes.

We have leveraged both metabolomics and transcriptomics of *Salmonella*-infected superspreader mice to define an important molecular mechanism of pathogen expansion within the GI tract. These results shed light on the metabolomic landscape within superspreaders and provide a holistic snapshot of complex host-pathogen interactions occurring in the GI tract, at the resolution of single metabolites. The metabolomic and transcriptomic datasets presented in this study will likely enable future studies of additional mechanisms that underlie the formation of superspreaders. Ultimately, this work contributes to the understanding of pathogen expansion within superspreader hosts, an area of study necessary for disease transmission control.

## STAR★Methods

### Key resources table

**Table:** 

REAGENT or RESOURCE	SOURCE	IDENTIFIER
**Bacterial and virus strains**		

*Salmonella enterica* serovar Typhimurium strain SL1344	Monack lab strain collection	N/A
*S.* Typhimurium Δ*orgA*Δ*ssaV:: Kan*^*R*^	Monack lab strain collection	N/A
*S.* Typhimurium Δ*caiTABCD:: Kan*^*R*^	This paper	N/A
*S.* Typhimurium Δ*araBAD:: Kan*^*R*^	Monack lab strain collection	N/A
*S.* Typhimurium Δ*orgA*Δ*ssaV*Δ*araBAD:: Kan*^*R*^	This paper	N/A
*S.* Typhimurium Δ*0148:: Kan*^*R*^	This paper	N/A
*S.* Typhimurium Δ*orgA*Δ*ssaV*Δ*0148:: Kan*^*R*^	This paper	N/A

**Chemicals, peptides, and recombinant proteins**		

LB Agar, Miller	Fischer Scientific	Cat# BP9724-2
LB Broth, Miller	Fischer Scientific	Cat# BP1426-500
Carbenicillin disodium salt	GoldBio	Cat# C-103-5
Penicillin- streptomycin	Thermo Fisher Scientific	Cat# 15-140-122
Kanamycin monosulfate	GoldBio	Cat# K-120-5
Chloramphenicol	GoldBio	Cat# C-105-5
L-(+)-Arabinose	Millipore Sigma	Cat# C973P47
NdeI	New England Bioloabs	Cat# R0111S
XhoI	New England Biolabs	Cat# R0146S

**Critical commercial assays**		

Phusion® High-Fidelity DNA Polymerase	New England BioLabs	Cat# M0530
MinElute PCR Purification Kit	Qiagen	Cat# 28004
RNeasy PowerMicrobiome Kit	Qiagen	Cat# 26000-50
Rneasy Mini Kit	Qiagen	Cat# 74106
Illumina® Stranded Total RNA Prep, Ligation with Ribo-Zero Plus	Illumina	Cat# 20040525
IDT® for Illumina® RNA UDIndexes Set A, Ligation	Illumina	Cat# 20040553
SuperScript III First-Strand Synthesis Kit	Thermo Fisher Scientific	Cat #18080051
FastStart Universal SYBR Green Master Mix	Sigma Aldrich	Cat #4913850001
Lipocalin-2 (LCN2) Mouse ELISA Kit	Invitrogen	Cat# EMLCN2
Qubit™ RNA High Sensitivity (HS), Broad Range (BR), and Extended Range (XR) Assay Kits	Thermo Fisher Scientific	Cat# Q10210

**Experimental models: Organisms/strains**		

129SvJ/X1	Jackson Laboratory	Cat# 000691
GF Swiss-Webster	Sonnenburg Lab	N/A
Rodent diet		
Standard diet	Envigo-Teklad	TD.2018
Polysaccharide-defined (arabinose-free) diet	Envigo-Teklad	TD.150689

**Oligonucleotides**		

Please see Table S2.	This study	

**Recombinant DNA**		

pKD4	Monack lab plasmid collection	N/A
pKD46	Monack lab plasmid collection	N/A
pCP20	Monack lab plasmid collection	N/A
pFOG	Dirk Bumann lab	N/A

**Software and algorithms**		

GraphPad Prism version 8.4.3 for MacOS	GraphPad Software Inc	https://www.graphpad.com/scientific-software/prism/
R(v4.0.2)	R Core Team	https://www.r-project.org/
RStudio(v1.3)	RStudio Team	https://www.rstudio.com/
tidyverse(v1.3.0)	Wickham et al., 2019^67^	https://www.tidyverse.org/
ggplot2(v3.3.2)	Wickham, 2016^68^	https://cran.r-project.org/web/packages/ggplot2/index.html
Nfcore/rnaseq pipeline development branch	Ewels et al.^69^	https://nf-co.re/rnaseq
DESeq2 v1.28.0	Love et al.^70^	https://bioconductor.org/packages/release/bioc/html/DESeq2.html
XNomial v1.0.4	William R. Engels, University of Wisconsin, Madison – Genetics Department	https://cran.r-project.org/web/packages/XNomial/vignettes/XNomial.html#use

**Deposited and generated data**		

Metabolomics	This paper	Data S1
Raw RNA seq data accession number: GSE207764^71^	This paper	https://www.ncbi.nlm.nih.gov/geo/query/acc.cgi?acc=GSE207764

### Resource availability

#### Lead contact

Further information and requests for resources and reagents should be directed to the Lead Contact, Denise M. Monack (dmonack@stanford.edu).

#### Materials Availability

This study did not generate new unique reagents.

### Experimental model and subject details

#### Ethics Statement

Experiments involving animals were performed in accordance with NIH guidelines, the Animal Welfare Act, and US federal law. All animal experiments were approved by the Stanford University Administrative Panel on Laboratory Animal Care (APLAC) and overseen by the Institutional Animal Care and Use Committee (IACUC) under Protocol ID 12826. Animals were housed in a centralized research animal facility accredited by the Association of Assessment and Accreditation of Laboratory Animal Care (AAALAC) International.

#### Mouse Strains and Husbandry

129X1/SvJ mice were obtained from Jackson Laboratories. Female mice (6-7 weeks old) were housed under specific pathogen-free conditions in filter- top cages that were changed bi-monthly by veterinary or research personnel. Sterile water and food were provided ad libitum. Mice were given at least one week to acclimate to the Stanford Animal Biohazard Research Facility prior to experimentation. Mouse age, sex, lineage, and source facility were tracked for all experiments and did not correlate with infection outcomes.

Swiss-Webster mice were used for gnotobiotic experiments, and the sterility of germ-free mice was verified through routine 16S PCR amplification and anaerobic culture of feces by gnotobiotic facility staff. Mice were maintained on a 12-h light/dark cycle at 20.5 °C at ambient humidity, fed ad libitum, and maintained in flexible film gnotobiotic isolators for the duration of all experiments. Mice were controlled for sex and age within experiments.

#### Bacterial Strains and Growth Conditions

*Salmonella enterica* serovar Typhimurium SL1344 strains were maintained aerobically on LB agar supplemented with 200 g/mL streptomycin (LB-strep), 200 g/mL streptomycin + 15 g/mL tetracycline (LB-strep-tet), or 200 g/mL streptomycin + 40 g/mL kanamycin (LB-strep-kan) and grown aerobically overnight at 37°C with aeration.

### Method details

#### Mouse Infections

Female mice were infected with 10^8^ CFU *S.* Typhimurium by drinking. To avoid adverse injury by gavage needle mice were scruffed while 20μl of the inoculum was expelled into the mouth and observed to confirm the culture was swallowed, as previously described.^72^ We monitored shedding and observed the same shedding dynamics in the feces and systemic organs of previously published work.^11^^,^^46^^,^^73^ For competitive index experiments, mice were infected with a 1:1 mixture of each *S.* Typhimurium strain by drinking, totaling 10^8^ CFU *S.* Typhimurium. 1-2 fecal pellets were collected and resuspended in 500μl PBS. To determine CFU/g feces, fecal pellets were weighed, serially diluted, and plated onto LB plates supplemented with appropriate antibiotics. Competitive Index (CI) values were calculated as previously described.^74^

For terminal experiments, mice were sacrificed at the indicated time points. Mice were euthanized with carbon dioxide. Sterile dissection tools were utilized to collect spleen, liver, mesenteric lymph nodes (MLN), small intestine, cecum, and colon. Tissues were collected in 1-3 mL PBS and homogenized. Homogenates were plated as serial dilutions on LB agar supplemented with 200 mg/mL of appropriate antibiotics to enumerate CFU/g tissue. Each experiment was conducted a minimum of 3 times to ensure reproducibility, unless otherwise stated in the figure legend.

#### Untargeted Metabolomics

##### Sample collection

Untargeted Metabolomic profiling on fecal samples was conducted using ultra-high-performance liquid chromatography-tandem mass-spectrometry by Metabolon Inc. (Morrisville, USA). In summary, fecal samples (2-3 fecal pellets) were collected before infection and on days 7,14, and 21 after infection (with 10^8^ CFU *S.* Typhimurium). Mice were single housed for the fecal pellet collection. Samples were immediately transferred to dry ice and subsequently stored at -80°C until sent to Metabolon, Inc for processing and analysis.

##### Ultrahigh Performance Liquid Chromatography-Tandem Mass Spectroscopy (UPLC-MS/MS)

All methods utilized a Waters ACQUITY ultra-performance liquid chromatography (UPLC) and a Thermo Scientific Q-Exactive high resolution/accurate mass spectrometer interfaced with a heated electrospray ionization (HESI-II) source and Orbitrap mass analyzer operated at 35,000 mass resolution. The sample extract was dried then reconstituted in solvents compatible to each of the four methods. Each reconstitution solvent contained a series of standards at fixed concentrations to ensure injection and chromatographic consistency. One aliquot was analyzed using acidic positive ion conditions, chromatographically optimized for more hydrophilic compounds. In this method, the extract was gradient eluted from a C18 column (Waters UPLC BEH C18-2.1x100 mm, 1.7 μm) using water and methanol, containing 0.05% perfluoropentanoic acid (PFPA) and 0.1% formic acid (FA). Another aliquot was also analyzed using acidic positive ion conditions; however, it was chromatographically optimized for more hydrophobic compounds. In this method, the extract was gradient eluted from the same afore mentioned C18 column using methanol, acetonitrile, water, 0.05% PFPA and 0.01% FA and was operated at an overall higher organic content. Another aliquot was analyzed using basic negative ion optimized conditions using a separate dedicated C18 column. The basic extracts were gradient eluted from the column using methanol and water, however with 6.5mM Ammonium Bicarbonate at pH 8. The fourth aliquot was analyzed via negative ionization following elution from a HILIC column (Waters UPLC BEH Amide 2.1x150 mm, 1.7 μm) using a gradient consisting of water and acetonitrile with 10mM Ammonium Formate, pH 10.8.

##### Data Extraction and Compound Identification

Raw data was extracted, peak-identified and QC processed using Metabolon’s hardware and software. Compounds were identified by comparison to library entries of purified standards or recurrent unknown entities. Metabolon maintains a library based on authenticated standards that contains the retention time/index (RI), mass to charge ratio (*m/z*), and chromatographic data (including MS/MS spectral data) on all molecules present in the library. Furthermore, biochemical identifications are based on three criteria: retention index within a narrow RI window of the proposed identification, accurate mass match to the library +/- 10 ppm, and the MS/MS forward and reverse scores between the experimental data and authentic standards. The MS/MS scores are based on a comparison of the ions present in the experimental spectrum to the ions present in the library spectrum. While there may be similarities between these molecules based on one of these factors, the use of all three data points can be utilized to distinguish and differentiate biochemicals. More than 3300 commercially available purified standard compounds have been acquired and registered into LIMS for analysis on all platforms for determination of their analytical characteristics. Additional mass spectral entries have been created for structurally unnamed biochemicals, which have been identified by virtue of their recurrent nature (both chromatographic and mass spectral).

##### Statistical analysis of metabolomic data

Metabolon, USA performed normalization to sample volume, log transformation, and imputation of missing values, if any, with the minimum observed value for each compound. Analysis by two-way ANOVA with repeated measures identified biochemicals exhibiting significant interaction and main effects for experimental parameters of shedding level, time point, and infection status. Standard statistical analyses were performed in ArrayStudio on log transformed data by Metabolon, USA. PCA was generated using log transformed metabolite levels. Pathway analysis was performed using the XNomial package v1.0.4 in R v4.0.2 for likelihood ratio test with Benjamini & Hochberg correction.

##### RNA Isolation.

RNA from cecal contents and feces was extracted using the QIAGEN RNeasy PowerMicrobiome Kit following manufacturer’s instructions. RNA from liquid cultures was extracted with the QIAGEN RNeasy Mini Kit following the QIAGEN supplementary protocol: purification of total RNA from bacteria. Three milliliters of culture were centrifuged at 6,000g for 10 min, resuspended in kit buffer RLT + beta-mercaptoethanol, and bead-beat using acid-washed glass beads for 5 min at 4°C. RNA was quantified using a Quibit and quality determined by an Agilent bioanalyzer.

##### RNA Library preparation and sequencing.

RNA libraries were prepared using the Illumina stranded total RNA prep ligation with ribo-zero plus kit. rRNA was depleted prior to library construction and confirmed with Agilent Bioanalyzer Prokaryote Total RNA Pico. RNA integrity was confirmed with Agilent Bioanalyzer Prokaryote Total RNA Pico. *In vivo* and *in vitro* libraries underwent quality control by Novogene Corporation Inc. Pulled *in vivo and in vitro* libraries were loaded onto separate lanes of an S4 flow cell and sequenced on an Illumina NovaSeq 6000 using the PE150 strategy. Data were unequally shared, 10.11G raw data (100.4 million paired reads) for each *in vivo* sample and 4.5G raw data (15 million paired reads) for each *in vitro* sample.

##### RNA-seq analysis

RNA-seq analysis was performed using the nf-core RNA-seq pipeline development branch version.^69^ Quality control was performed using FastQC v0.11.9. Adapters and low-quality reads were filtered by TrimGalore! with default parameters v0.6.5. Quality reads were aligned to *S*. Tm strain SL1344 genome using HiSAT2 v2.2.1.^75^ Stringtie was used to assemble transcript alignments and for quantification v2.1.4.^76^ Reads were further processed in Deseq2^70^ for differential expression analysis. Results were plotted in Graph pad prism v8.1.2 or R using ggplot2. Biocyc Pathway Tools was used for pathway enrichment analysis.^77^

##### Bacterial Strain Construction

Genes in the SL1344 genome were targeted for deletion following ƛ-red mutagenesis protocol.^78^ Briefly, primers were used to amplify the kanamycin resistance cassette of pKD4, and PCR products were purified. *S.* Tm strains 14028 containing pKD46 were grown at 30ºC, 200 rpm to mid-log phase, L-arabinose was added to a final concentration of 50 mM and bacteria were incubated for an additional hour. 1-5 μg of purified PCR product was added to washed and concentrated bacteria. Bacteria were electroporated and recovered in 30 ºC SOC broth for 3 hours with shaking. Cultures were plated on antibiotic selection plates and grown overnight at 42 ºC to cure cells of the pKD46 plasmid. Colonies were PCR verified for the kanamycin resistance insertion and endogenous gene disruption. Removal of the pKD46 plasmids was confirmed by a loss of carbenicillin resistance. P-22 phage transduction was used to introduce desired gene knockouts to SL1344. H-5 phage was used to confirm final stains as lysogen-free. Final SL1344 strains were confirmed through PCR. To complement the *S*. Tm^*0148*^ strain, we used a duel-negative selection approach recently optimized for *Salmonella*.^79^ Briefly, *S*. Tm^*0148::0148*^ was generated by amplifying the 0148 gene with 1000 bp upstream and downstream of the gene, cloned into the provided pFOG vector, transformed into an *E. coli* donor strain, and conjugated with *S*. Tm^*0148*^*.*

##### Custom research diets

Animals were maintained on standard diet (SD, Envigo TD.2018, Teklad Global 2018 rodent diet) for at least one week prior to experimentation. If mice were switched to an arabinose-free diet (polysaccharide-defined: custom diet, Envigo TD.150689 all carbohydrate and fiber sources replaced with dextrose monohydrate), this occurred at time of infection.

##### L-Arabinose Quantification

Feces were weighed, homogenized in 10% ethanol, and spun at 14,000g for 5 minutes. Supernatant was sent to UCSD’s Glycoanalytics Core. There, Samples were centrifuged at 10,000g for 5 minutes and spin filtered using prewashed 3K spin filtration unit (Centrifugal device, Pall, Life Sciences, Part No. OD003C34). Flow through was spiked with 1.0μg of Inositol as internal standard and lyophilized. Dried material was reduced using sodium borohydride in presence of 1M ammonium hydroxide, overnight at room temperature. Excess reducing agent was neutralized on ice bath using 30% aqueous acetic acid solution. Samples were then dried by nitrogen flush and co-evaporated with 9:1 methanol: acetic acid mixture (3 times) followed by anhydrous methanol (3 times). Finally, the samples were acetylated using mixture of pyridine and acetic anhydride (1:1 v/v) and alditol acetylate of monosaccharides were analyzed by GCMS (Agilent Technologies, 7820 GC System attached with 5975-MSD). Restek-5ms column (30m x 0.25mm x 0.25μm) was used for profiling of the monosaccharides, Ultrapure He was used as carrier gas in split less mode at a flow rate of 1.2mL/min. Quantification of monosaccharides were done based on the response factors obtained from known amount of standard mixture.

##### Lipocalin ELISA

Feces were collected from mice, weighed, and stored at -80 ºC until protein quantification could be performed. Samples were homogenized by bead beating and protein was assessed using a Lipocalin-2 (LCN2) Mouse ELISA Kit (Invitrogen) according to manufacturer’s instructions.

##### qRT-PCR

Spleen and colon tissues were harvested from mice and stored at -80 ºC until RNA extractions were performed. Tissues were homogenized by bead beating and RNA was isolated using an RNeasy Mini Kit (Qiagen). RNA concentration was determined with a Nanodrop spectrophotometer and samples were stored at -80 ºC. cDNA was synthesized from RNA samples using a SuperScript III First-Strand Synthesis Kit (Thermo Fisher Scientific) and stored at -20 ºC. To assess expression of inflammatory genes, cDNA and gene-specific primers were used in combination with FastStart Universal SYBR Green Master Mix (Sigma Aldrich) to amplify transcripts.

### Quantification and Statistical Analysis

All statistical analyses were performed in R or Prism v. 8.1.2 (GraphPad) and visualized with ggplot2 and Prism v. 8.1.2 (GraphPad). Statistical significance of all CFU counts were determined by 2-way ANOVA in Prism v. 8.1.2 (GraphPad). Statistical significance of time-course competitive index infection data was determined by Mann-Whitney U test in Prism v. 8.1.2 (GraphPad).

## Data Availability

• Untargeted metabolomics data are attached as a supplementary file to this manuscript (Data S1). • Raw RNA-seq data discussed in this study have been deposited in NCBI's Gene Expression Omnibus and are accessible through GEO Series accession number GSE207764 (https://www.ncbi.nlm.nih.gov/geo/query/acc.cgi?acc=GSE207764). • Code is available at https://github.com/sruddle/superspreader • Any additional information required to reanalyze the data reported in this work paper is available from the Lead Contact upon request.

## Supplemental information

Document S1. Figures S1–S5 and Tables S1 and S2

Data S1. Scaled metabolite levels and significant biochemicals of stool metabolomics, related to Figure 1

Document S2. Article plus supplemental information
