# Supplementary material for: Salmonella-liberated dietary L-arabinose promotes expansion in superspreaders
Source: Cell Host Microbe. 2023 Mar 8;31(3):405–417.e5. doi: 10.1016/j.chom.2023.01.017 (PMC10016319; doi:10.1016/j.chom.2023.01.017)
Supplement: Document S1. Figures S1–S5 and Tables S1 and S2 [file mmc1.pdf]

Cell Host & Microbe, Volume *31*

Supplemental information

Salmonella-liberated dietary L-arabinose promotes expansion in superspreaders

Sarah J. Ruddle, Liliana M. Massis, Alyssa C. Cutter, and Denise M. Monack

Supplemental figure 1

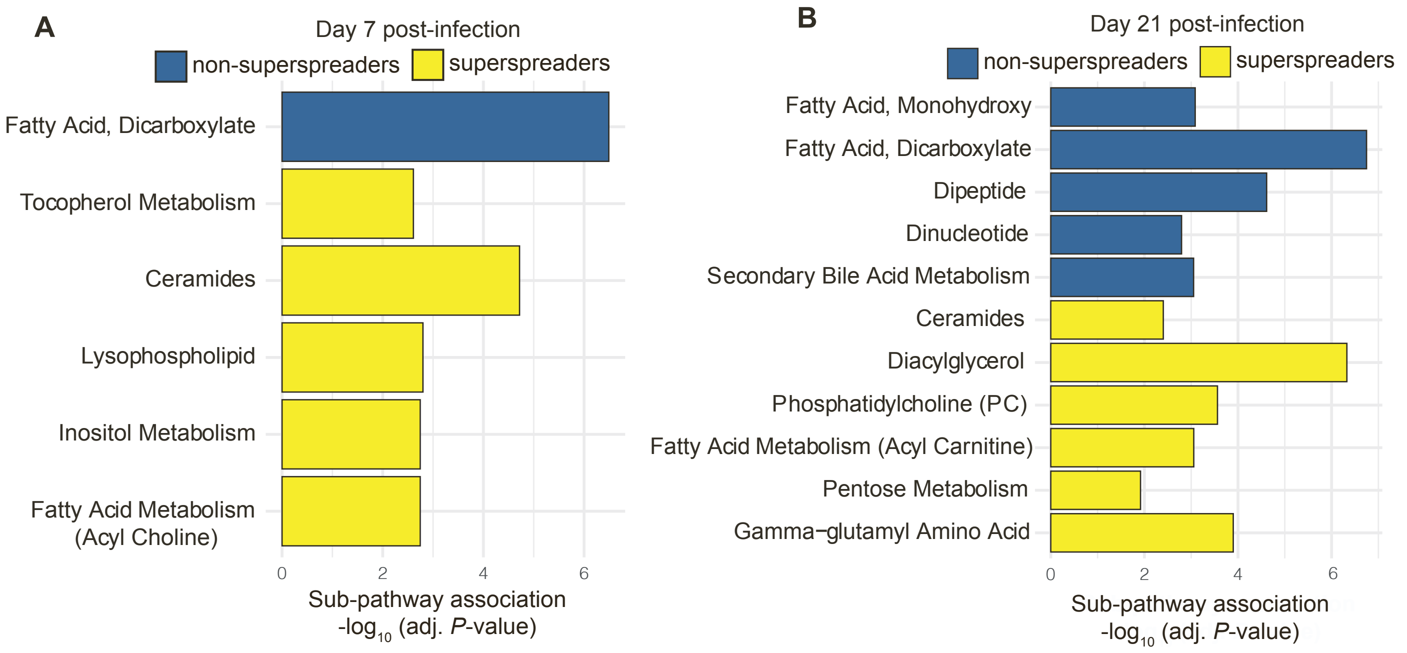

Supplemental figure 2

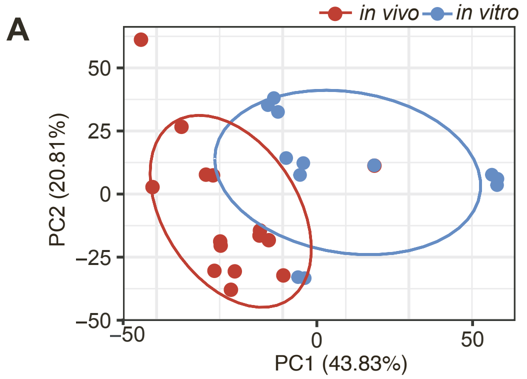

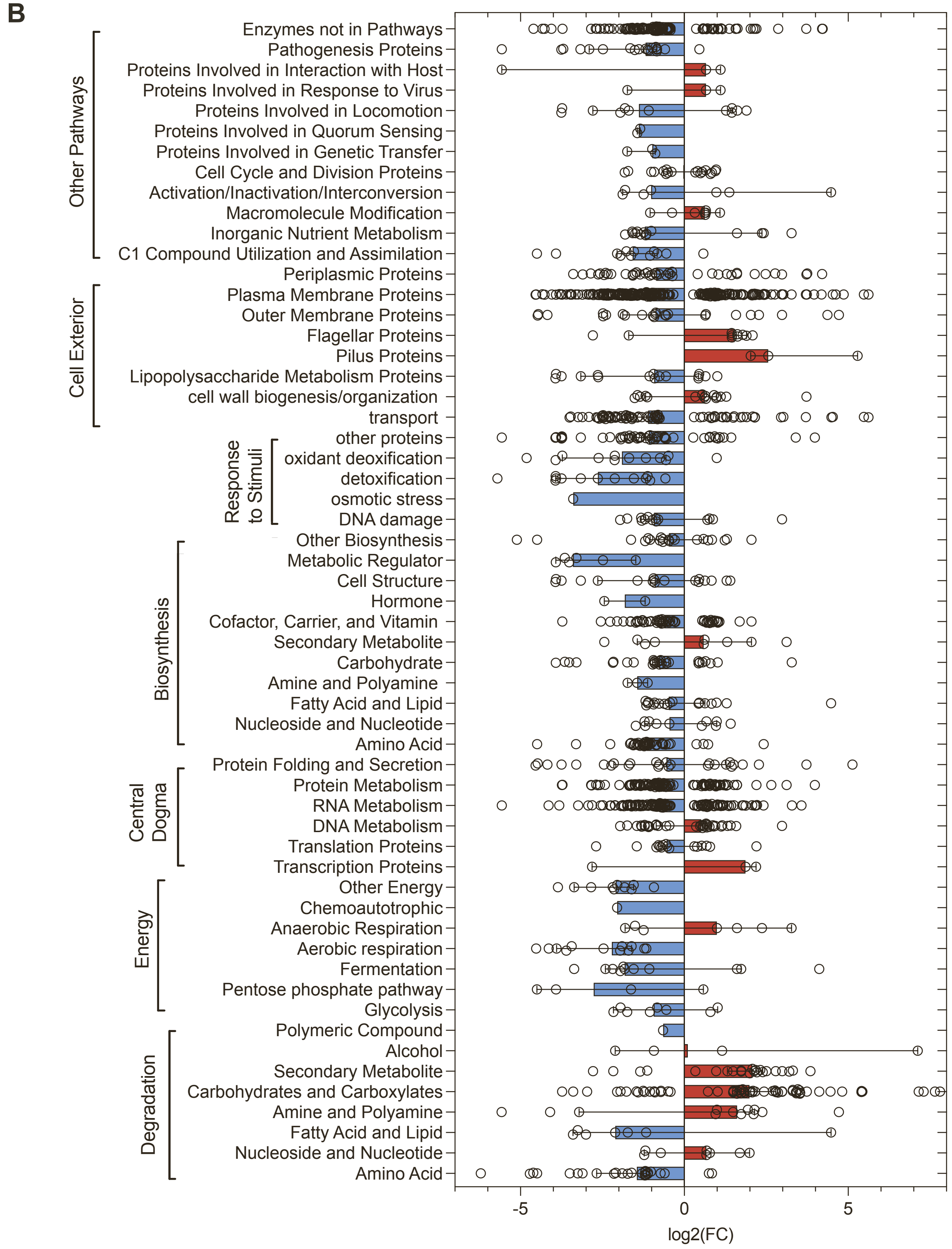

Supplemental figure 3

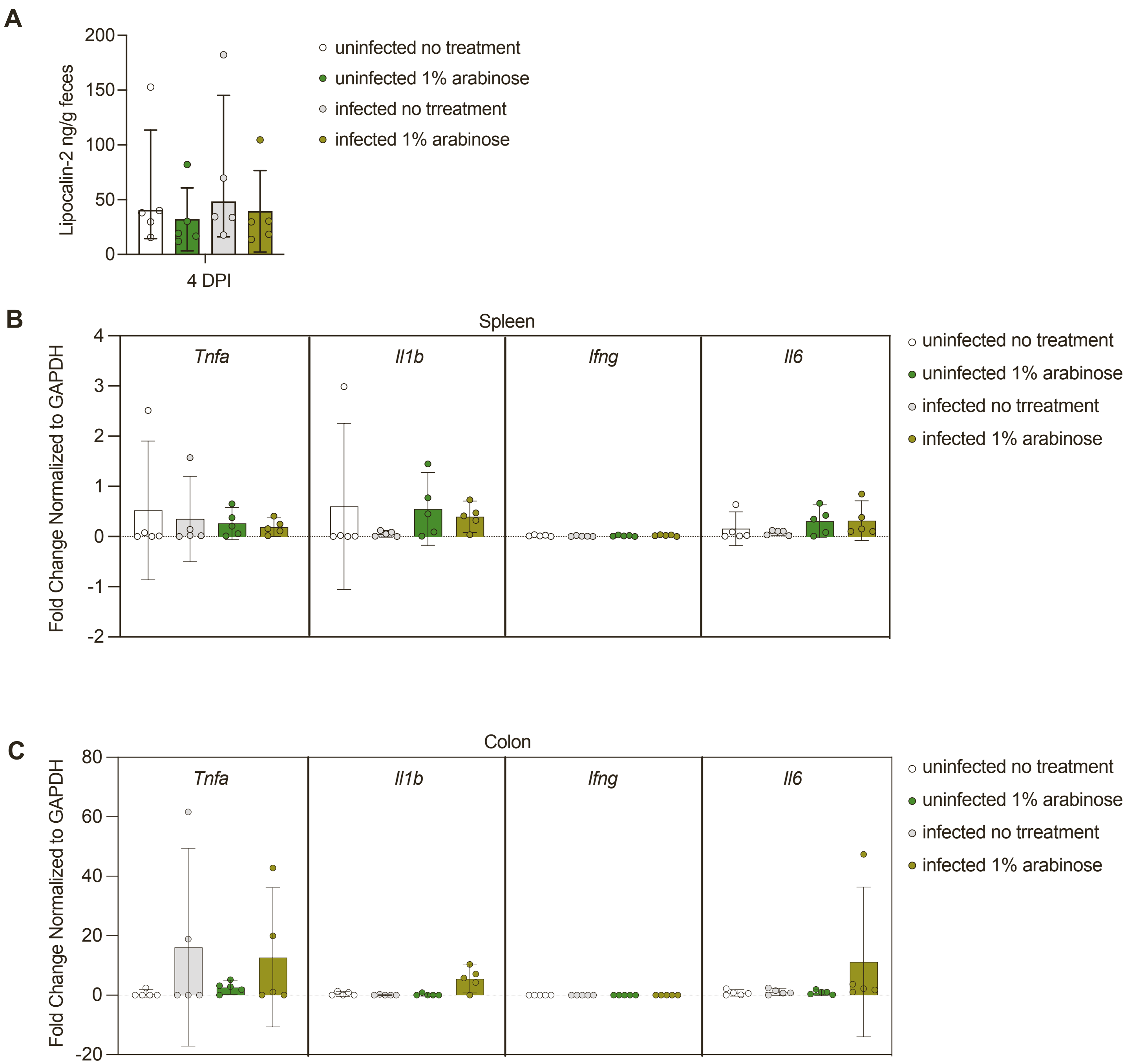

Supplemental figure 4

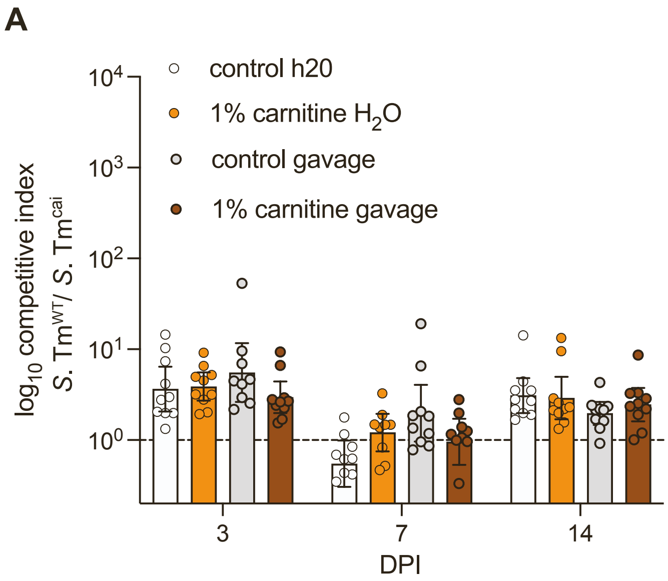

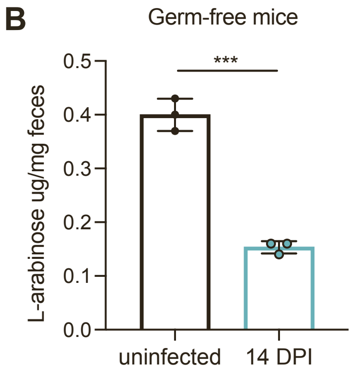

Supplemental figure 5

A

```
1          11         21         31         41        50
|          |          |          |          |         |
MTTSLNSRRL WLHRLCALLL GTGSAL---- ------VQAE NPIFTDVFTA   40 C. japonicus
MRKKC-SVCL WILVLLLSCL SGKSAYAATS TTIAKHIGNS NPLIDHHLGA   49 B. subtilis
M-RRL-TVRL FTAVLAALAL LTMGTPAHAT APASPSVTFT NPLAEKR--A   46 S. avermitilis
M--------- ---------- ---------- ------ANWP NPFIEQR--A   13 S. Typhimurium

DPAALVH-KG RVYLYAGRDE APDNT----- --TFFVMNEW LVYSSDDMAN   82 C. japonicus
DPVALTY-NG RVYIYMSSDD YEYNSNGTIK DNSFANLNRV FVISSADMVN   98 B. subtilis
DPHIFKHTDG Y-YYFTATVP -EYDRIVLRR ATTLQGLA-- ---TAPETTI   89 S. avermitilis
DPFILR--DG SDYYFIASVP -EYDRLEIRR ANSLEGLR-- ---AADPVVV   55 S. Typhimurium

WEAHGPGLR- -AKDFTWAKG DA------WA SQVI--ERNG --KFYWYVTV  120 C. japonicus
WTDHGAIPVA GANGANGGRG IAKWAGASWA PSIAVKKING KDKFFLYFA-  147 B. subtilis
WTKHASGVM- ---------- ----GAHIWA PEIH--FIDG --KWYVYFAA  120 S. avermitilis
WRKPESGPM- ---------- ----SQLIWA PEMH--RING --KWYIYFAA   86 S. Typhimurium

RHDD------ --TKPGFAIG VAVGDSPIGP FKDALGKALI TNDMTTDTPI  162 C. japonicus
---------- ---NSGGGIG VLTADSPIGP WTDPIGKPLV TPSTPGMSGV  184 B. subtilis
GSTS-----D VWAIRMYVLE SGAANPLTGS W--------- TEKGQIATPV  156 S. avermitilis
THTQALDKLG MFQHRMFALE CADADPLTGK W--------- TEKGQIKTPF  127 S. Typhimurium

DWDDIDPSVF IDDDGQAYLF WGNTRP---- ---------- -----RYAKL  193 C. japonicus
VW-LFDPAVF VDDDGTGYLY AGGGVPGVSN PTQ---GQWA NPKTARVIKL  230 B. subtilis
SSFSLDATTF VVN-GVRHLA WAQRNPAEDN NTSLFIAKMA NPWT------  199 S. avermitilis
DTFALDATTF YHQ-GKQWYL WAQKAPDIAG NSNIYLAELE NPWT------  170 S. Typhimurium

KKNMVELDGP IRAIEGLPEF T--------- EAIWVHKYQD NYYLSY----  230 C. japonicus
GPDMTSVVGS ASTIDA-PFM F--------- EDSGLHKYNG TYYYSY----  266 B. subtilis
------ISGT PTEISQ-PTL SWETVGYKVN EGPAVIQHGG KVFLTYSASA  242 S. avermitilis
------IKGE PVRLSK-PEY DWECRGFWVN EGPAVVVHGD KLFISYSASA  213 S. Typhimurium
```

B

```
SL1344                    1 MANWPNPFIEQRADPFILRDGSDYYFIASVPEYDRLEIRRANSLEGLRAADPVVVWRKPESGPMS  65
S. Typhimurium D23580     1 MANWPNPFIEQRADPFILRDGSDYYFIASVPEYDRLEIRRANSLEGLRAADPVVVWRKPESGPMS  65
S. Typhimurium 4,[5],12:i:- 1 MANWPNPFIEQRADPFILRDGSDYYFIASVPEYDRLEIRRANSLEGLRAADPVVVWRKPESGPMS  65
S. Dublin ATCC 39184      1 MANWPNPFIEQRADPFILRDGSDYYFIASVPEYDRLEIRRADSLEGLRAADPVVVWRKPESGPMS  65
S. Paratyphi A ATCC 9150  1 MANWPNPFIEQRADPFILRDGSDYYFIASVPEYDRLEIRRANSLEGLRAADPVVVWRKPKTGPMS  65
S. Typhi Ty2              1 MANWPNPFIEQRADPFILRDGSDYYFIASVPEYDRLEIRRADSLEGLRAADPVVVWRKPESGPMS  65

SL1344                   66 QLIWAPEMHRINGKWYIYFAATHTQALDKLGMFQHRMFALECADADPLTGKWTEKGQIKTPFDTF  130
S. Typhimurium D23580    66 QLIWAPEMHRINGKWYIYFAATHTQALDKLGMFQHRMFALECADADPLTGKWTEKGQIKTPFDTF  130
S. Typhimurium 4,[5],12:i:- 66 QLIWAPEMHRINGKWYIYFAATHTQALDKLGMFQHRMFALECADADPLTGKWTEKGQIKTPFDTF  130
S. Dublin ATCC 39184     66 QLIWAPEMHRINGKWYIYFAATHTQALDKLGMFQHRMFALECADADPLTGKWTEKGQIKTPFDTF  130
S. Paratyphi A ATCC 9150 66 QLIWAPEMHRINGKWYIYFAATHTQALDKLGMFQHRMFALECADADPLTGKWTEKGQIKTPFDTF  130
S. Typhi Ty2             66 QLIWAPEMHRINGKWYIYFAATHTQALDKLGMFQHRMFALECADADPLTGKWTEKGQIKTPFDTF  130

SL1344                  131 ALDATTFYHQGKQWYLWAQKAPDIAGNSNIYLAELENPWTIKGEPVRLSKPEYDWECRGFWVNEG  195
S. Typhimurium D23580   131 ALDATTFYHQGKQWYLWAQKAPDIAGNSNIYLAELENPWTIKGEPVRLSKPEYDWECRGFWVNEG  195
S. Typhimurium 4,[5],12:i:- 131 ALDATTFYHQGKQWYLWAQKAPDIAGNSNIYLAELENPWTIKGEPVRLSKPEYDWECRGFWVNEG  195
S. Dublin ATCC 39184    131 ALDATTFYHQGKQWYLWAQKAPDIAGNSNIYLAELENPWTIKGEPVRLSKPEYDWECRGFWVNEG  195
S. Paratyphi A ATCC 9150 131 ALDATTFYHQGKQWYLWAQKAPDIAGNSNIYLAELENPWTIKGEPVRLSKPEYDWECRGFWVNEG  195
S. Typhi Ty2            131 SLDATTFYHQGKQWYLWAQKAPDIAGNSNIYLAELENPWTLKGEPVRLSKPEYDWECRGFWVNEG  195

SL1344                  196 PAVVVHGDKLFISYSASATDENYCMGLLWINVNDDPRDPANWHKSPRPVFTTSYENRQYGPGHNS  260
S. Typhimurium D23580   196 PAVVVHGDKLFISYSASATDENYCMGLLWINVNDDPRDPANWHKSPRPVFTTSYENRQYGPGHNS  260
S. Typhimurium 4,[5],12:i:- 196 PAVVVHGDKLFISYSASATDENYCMGLLWINVNDDPRDPANWHKSPRPVFTTSYENRQYGPGHNS  260
S. Dublin ATCC 39184    196 PAVVVHGDKLFISYSASATDENYCMGLLWINVNDDPRDPANWHKSPRPVFTTSYENRQYGPGHNS  260
S. Paratyphi A ATCC 9150 196 PAVVVHGDKLFISYSASATDENYCMGLLWINVNDDPLTPQNWHKSPRPVFTTSYENRQYGPGHNS  260
S. Typhi Ty2            196 PAVVVHGDKLFISYSASATDENYCMGLLWINVNDDPRDPANWHKSPRPVFTTSYENRQYGPGHNS  260

SL1344                  261 FTQTPEGENVLVYHARNYTEIEGDPLYDPNRHTRLKRVRWDENGMPDFGVPPADTI  316
S. Typhimurium D23580   261 FTQTPEGENVLVYHARNYTEIEGDPLYDPNRHTRLKRVRWDENGMPDFGVPPADTI  316
S. Typhimurium 4,[5],12:i:- 261 FTQTPEGENVLVYHARNYTEIEGDPLYDPNRHTRLKRVRWDENGMPDFGVPPADTI  316
S. Dublin ATCC 39184    261 FTQTPEGEDVLVYHARNYTEIEGDPLYDPNRHTRLKRVRWDENGMPDFGVPPADTI  316
S. Paratyphi A ATCC 9150 261 FTQTPEGEDVLVYHARNYTEIEGDPLYDPNRHTRLKRVRWDENGMPDFGVPPADTI  316
S. Typhi Ty2            261 FTQTSEGEDVLVYHARNYTEIEGDPLYDPNRHTRLKRVRWDENGMPDFGVPPADTI  316
```

Supplemental figure 1. *S.* Tm superspreaders and non-superspreaders have differentially enriched metabolic pathways at 7 and 14 DPI

Corresponds with figure 1

A) Differential pathway enrichment of non-superspreaders and superspreaders at 7 DPI with multiple hypothesis correction (Likelihood ratio with Benjamini & Hochberg correction).

B) Differential pathway enrichment of non-superspreaders and superspreaders at 21 DPI with multiple hypothesis correction (Likelihood ratio with Benjamini & Hochberg correction).

Supplemental figure 2. *S.* Tm genetic pathways enriched *in vivo* and *in vitro*

Corresponds with figure 2

A) Log2 fold differences of differentially enriched genetic pathways (adjusted p-value < 0.05) *in vitro* (blue) and *in vivo* (red). Each data point represents single gene within each sub pathway (y-axis). Sub pathways are annotated with corresponding major pathways (brackets along y-axis)

B) Principal component analysis of *in vivo* and *in vitro* transcriptomes. *In vivo* and *in vitro* transcriptomes separate along PC1.

Supplemental figure 3: Exogenous dietary L-arabinose does not induce inflammation

Corresponds with figure 4. The data in this figure are from a single mouse experiment.

A) Lipocalin-2 levels detected in feces of the indicated groups 4-days post treatment (diet and/or infection) by Elisa. n=5 mice

B) qRT-PCR of mRNA extracted from spleens from indicated groups 5 days post treatment. n=5 mice.

C) qRT-PCR of mRNA extracted from colons from indicated groups 5 days post treatment. n=5 mice

Supplemental figure 4: Carnitine supplementation does not confer a competitive advantage to WT *S.* Tm and L-arabinose concentrations decrease during infection in germ-free mice

Corresponds with figure 4

A) 129X1/SvJ mice were inoculated with an equal mixture of *S.* Tm^{WT} and *S.* Tm^{cai}. Each dot represents data from one animal (biological replicate). Bars represent +- SEM. *p < 0.05, ** p < 0.01. Kruskal-Wallis. No difference across groups. N=10 per group.

B) L-Arabinose concentration in the germ-free mouse feces was quantified by liquid LC/MS. Students T test, *** p < 0.001

Supplemental figure 5. *S.* Tm arabinofuranosidase has conserved functional residues with previously characterized arabinofuranosidases

Corresponds with figure 5

A) Amino acid sequence alignment of STM0148 (grey highlight) against three characterized arabinofuranosidase amino acid sequences. Red rectangles show conserved critical residues for enzyme function.

B) Amino acid sequence alignment of STM0148 against five other *Salmonella* serovars'' arabinofuranosidase amino acid sequences. Red rectangles show conserved critical residues for enzyme function. Pink highlight indicates amino acid differences compared to SL1344 (first strand).

Table S1: Related to Figure 5. SL1344 predicted glycoside hydrolases

Gene ID	Cazyme family		Reference accession
SL1344_0018	GH18		CBW16119.1
SL1344_3087	GH23		CBW19186.1
SL1344_0016	GH108		CBW16117.1
SL1344_0042	GH31		CBW16143.1
SL1344_0148	GH43		CAB89837.1
SL1344_0234	GH19		CBW16336.1
SL1344_0255 (MltD)	GH23		CBW16357.1
SL1344_0396	GH13		CBW16496.1
SL1344_0966 (NucD)	GH24		CBW17063.1
SL1344_1119 (FlgJ)	GH73		CBW17215.1
SL1344_1146 (NagZ)	GH3		CBW17242.1
SL1344_1188	GH33		CBW17283.1
SL1344_1251 (CelF)	GH4		CBW17347.1
SL1344_1488 (GlgX)	GH13		CBW17583.1
SL1344_1489	GH13		CBW17584.1
SL1344_1490	GH13		CBW17585.1
SL1344_1724 (TreA)	GH37		CBW17819.1
SL1344_1727 (MltE)	GH23		CBW17822.1
SL1344_1846	GH105		CBW17940.1
SL1344_1892 (AmyA)	GH13		CBW17987.1
SL1344_1956	GH19		CBW18053.1
SL1344_2144 (BglX)	GH3		CBW18240.1
SL1344_2529 (YfhD)	GH23		CBW18629.1
SL1344_2576	GH24		CBW18677.1
SL1344_2686 (Nucd2)	GH24		CBW18788.1
SL1344_2811 (MltB)	GH103		CBW18909.1
SL1344_2857 (IagB)	GH23		CBW18955.1
SL1344_2968 (MltA)	GH102		CBW19067.1
SL1344_3027 (BglA)	GH1		CBW19126.1
SL1344_3480 (MalQ)	GH77		CBW19575.1
SL1344_3504 (GlgX)	GH13		CBW19598.1
SL1344_3505 (GlgB)	GH13		CBW19599.1
SL1344_3568 (TreF)	GH37		CBW19662.1
SL1344_3570 (fragment)	GH24		CBW19664.1
SL1344_3582 (YhjM)	GH8		CBW19676.1
SL1344_3628 (BaX)	GH73		CBW19721.1
SL1344_3629 (MalS)	GH13		CBW19722.1
SL1344_3644	GH127		CBW19737.1
SL1344_3715	GH31		CBW19809.1
SL1344_3740	GH1		CBW19832.1

SL1344_3767	GHnc		CBW19857.1
SL1344_3965	GH31		CBW20055.1
SL1344_4153	GH23		CBW20241.1
SL1344_4235	GH4		CBW20321.1
SL1344_4358	GH30		CBW20445.1
SL1344_4384 (TreC)	GH13		CBW20472.1
SL1344_4509 (Slt)	GH23		CBW20598.1
SL1344_P2_0087 (PilT)	GH23		CCF76895.1

Table S2: Related to STAR Methods. Oligonucleotides used in this study

**Primer name: description**	**Sequence 5’-3’**	**Source**
SR114: caiTABCD forward lambda	AAATCGGGAATTGAACCGAAGGTTTTTTTCCGCC ATTAAGTGTAGGCTGGAGCTGCTTC	IDT
SR115: caiTABCD reverse lambda	CGGATCGCGTTTTTCGGCAAATGCCTGCGGTCCTT CGAGCCATATGAATATCCTCCTTAG	IDT
SR116: caiTABCD forward verification	CTGGCTCAACAATATTGAACGC	IDT
SR117: caiTABCD reverse verification	GCGAGTGGGCCAATATAAACAC	IDT
SR118: caiTABCD reverse/internal verification	CGCGATGGTGTTATACGCC	IDT
SR119: araBAD forward verification	GACCAGGACGACAGAGCTTCC	IDT
SR120: araBAD reverse verification	CAGATTCATCAACGCGCCC	IDT
SR121: araBAD reverse-internal verification	GCGTCAGGGTATAGCTGCTTTCATACTC	IDT
SR148: 0148 lamda forward	AAACCCGTTTATTGAACAACGTGCCGATCCGTTTA TTTTAGTGTAGGCTGGAGCTGCTTC	IDT
SR149: 0148 lamda reverse	TGGCGGCACGCCAAAATCAGGCATCCCGTTTTCGT CCCAGCATATGAATATCCTCCTTAG	IDT
SR150: 0148 forward verification primer	CGGCGTTGGCTATCTGATTA	IDT
SR151: 0148 reverse verification primer	CAATATCAGGTGCTCACACGTCTG	IDT
SR150: 0148 reverse-internal verification primer	CTGGGAATGTTCCAGCATCG	IDT
Mouse IFNγ qPCR forward	AGCGGCTGACTGAACTCAGATTGTAG	IDT
Mouse IFNγ qPCR reverse	GTCACAGTTTTCAGCTGTATAGGG	IDT
Mouse *GAPDH* qPCR F primer	ACAGTCCATGCCATCACTGCC	IDT
Mouse *GAPDH* qPCR R primer	GCCTGCTTCACCACCTTCTTG	IDT
Mouse TNFα qPCR F primer	GATCGGTCCCCAAAGGGATG	IDT
Mouse TNFα qPCR R primer	TGGTTTGTGAGTGTGAGGGTC	IDT
Mouse IL-6 qPCR F primer	TCCAGTTGCCTTCTTGGGAC	IDT

Mouse IL-6 qPCR R primer	AGTCTCCTCTCCGGACTTGT	IDT
Mouse IL-1β qPCR F primer	AGCTTCCTTGTGCAAGTGTCT	IDT
Mouse IL-1β qPCR R primer	GACAGCCCAGGTCAAAGGTT	IDT
Pfog forward	CGATTCGAATTCTATCGATAAGCTTGATATCGCGACAAG	IDT
Pfog reverse	CGATTCGGATCCCGCTCTAGAACTAGTGGATCGGATAAATC	IDT
0148_up_forward	CGACAAGTGAATCCAATAT	IDT
0148_up_reverse	CTTTTGGATCGTTAGTGATCACGCCCTGGCATTATCGTCTGTC	IDT
0148_insert_forward	CGTGATCACTAACGATCCAAAAG	IDT
0148_insert_reverse	CAGTTTGCGGGAAGACTTTCAC	IDT
